# Citrus Carotenoid Extracts Promote ROS Accumulation and Induce Oxidative Stress to Exert Anti-Proliferative and Pro-Apoptotic Effects in MDA-MB-231 Cells

**DOI:** 10.3390/antiox13030264

**Published:** 2024-02-22

**Authors:** Juanjuan Wei, Zimao Ye, Yurong Li, Yi Li, Zhiqin Zhou

**Affiliations:** 1 Key Laboratory of Agricultural Biosafety and Green Production of Upper Yangtze River (Ministry of Education), College of Horticulture and Landscape Architecture, Southwest University, Beibei District, Chongqing 400715, China; wei000319@email.swu.edu.cn (J.W.); yezimao@email.swu.edu.cn (Z.Y.); yellorlyr@email.swu.edu.cn (Y.L.); 2 Zhejiang Citrus Research Institute, Taizhou 318020, China; liyi1991@email.swu.edu.cn; 3 The Southwest Institute of Fruits Nutrition, Banan District, Chongqing 400054, China

**Keywords:** carotenoids, citrus, reactive oxygen species (ROS), apoptosis, oxidative stress

## Abstract

Citrus fruits are economically significant crops worldwide, as they contain various bioactive compounds that possess health-promoting properties. Carotenoids, as the most important component in citrus, exhibit notable pharmacological activities, such as antioxidation and anticancer, which make carotenoids valuable in the prevention and treatment of breast cancer. In this study, after treatment with carotenoid extracts from XiYou (XY) and ZaoHongQiCheng (ZH), we evaluated the cytotoxicity, apoptosis, antioxidant system, and oxidative stress induced by ROS overproduction and MMP damage in MDA-MB-231 cells. The analysis confirmed that cell proliferation was inhibited in a concentration-dependent manner, accompanied by G0/G1 arrest and cell apoptosis. XY and ZH promoted the accumulation of ROS, decreased MMP, increased malondialdehyde (MDA) levels, consumed glutathione (GSH), and reduced the activity of antioxidant enzymes (peroxidase (POD), catalase (CAT), glutathione reductase (GR), and superoxide dismutase (SOD)). Meanwhile, XY and ZH induced apoptosis through the mitochondrial pathway by significantly upregulated *P53*, *BAX*, *caspase-3*, *caspase-7*, and *caspase-9* gene expression levels and downregulated *Bcl-2*. Carotenoid-rich extracts were found to cause oxidative stress by enhancing ROS production through their pro-oxidative potential, and the aggravation of oxidative processes promotes apoptosis in MDA-MB-231 cells. These results indicate that citrus carotenoids can be used as potential pro-oxidants and have the potential to be developed into products for the prevention or treatment of breast cancer.

## 1. Introduction

Cancer has become one of the main triggers threatening people’s health, with approximately 9.6 million deaths worldwide in 2018. Breast cancer is a heterogeneous tumor and is the most frequently diagnosed cancer in women. It significantly affects the longevity and quality of life of women worldwide [1]. According to statistics, the incidence of breast cancer has increased significantly, and one-third of the diagnosed cancer cases in women are breast cancer [1,2]. Previous studies have shown that breast cancer can be categorized into different subtypes, such as luminal A, luminal B, basal-like, and HER2 subtypes. However, about 15–20% of breast cancer patients are diagnosed with triple-negative breast cancer (TNBC), which is characterized by estrogen receptor (ER-) negative, progesterone receptor (PR-) negative, and human epidermal growth factor receptor 2 (HER2-) negative [3]. Unlike other breast cancer subtypes, TNBC has no established treatment targets and can only be treated with chemotherapy drugs, resulting in little progress and significant therapeutic challenges [4]. At present, the existing therapies for breast cancer include surgical intervention, chemotherapy, radiotherapy, and hormone therapy, which have the drawbacks of drug resistance and side effects [5]. Therefore, there is an urgent requirement for effective treatment strategies for breast cancer.

In recent years, therapies based on bioactive compounds in natural plants and plant derivatives have gained popularity as potential alternative or complementary therapies to conventional medicine, particularly in the treatment of cancer [6]. Citrus has received extensive attention from researchers due to its high biological activities and health-promoting benefits, such as antioxidant, anti-cancer, anti-aging, neuropharmacological, cardio-protective, and hepato-protective [7,8,9,10,11,12]. The World Health Organization states that a good diet rich in fruits and vegetables is important in preventing cancer development, which is consistent with a large number of studies that identify a range of secondary metabolites of edible plants as bioactive compounds with the potential to prevent cancer, including carotenoids [13]. Carotenoids are tetraterpenoids, a class of natural pigments synthesized mainly in plants, fungi, algae, and bacteria, which are beneficial nutrients with protective and therapeutic effects on human health [14]. However, humans are unable to synthesize carotenoids on their own and must obtain them through dietary intake, primarily from fruits and vegetables [15]. To date, approximately 700 carotenoids have been identified, which are mainly divided into two categories according to their structural characteristics: oxygenated derivatives of carotenes and hydrocarbon carotenoids [16]. Perhaps precisely because of the wide variety and selectivity of carotenoids, they have become potential candidates for chemoprevention and chemotherapy of breast, colorectal, lung, and prostate cancers in recent years [13]. Citrus has become an important source of dietary carotenoid supplements due to its great diversity in carotenoid composition and concentration [17,18].

Given the evidence demonstrating that carotenoid intake is significantly negatively correlated with breast cancer [19], studies have shown that carotenoids exert significant anti-cancer effects by regulating multiple molecular mechanisms, such as oxidative stress [20], cell cycle arrest [21], anti-metastasis [22], pro-apoptosis [23], anti-proliferation [24], multidrug resistance [25], and signal transduction pathways. The preventive anticancer activity of carotenoids is mainly related to their high antioxidant properties, which can reduce ROS-induced DNA damage [26]. However, the anticancer properties of carotenoids are associated with pro-oxidant activity and cellular mechanisms [27]. The pro-oxidative activity of carotenoids causes the accumulation of ROS in cancer cells, resulting in oxidative stress, which limits the progression and metastasis of cancer [28,29]. Pro-oxidants induce apoptosis by enhancing ROS signaling pathways and/or diminishing the antioxidant mechanism of cancer cells [30]. Interestingly, carotenoids act as pro-oxidant molecules under an unbalanced cellular redox state, high oxygen tension, and high concentrations [31]. Currently, pro-oxidants have emerged as promising drug candidates for selectively targeting tumor cells [30]. Therefore, the role of carotenoids as pro-oxidants, especially from natural and edible plants, has broad prospects in cancer therapy. In fact, some carotenoids, including β-carotene, lycopene, and lutein, have anti-proliferative and pro-apoptotic effects on breast cancer [32]. Four xanthophylls in lettuce have a significant inhibitory effect on cervical cancer and lung cancer cells [33]. The β-carotene isolated from Spinacia oleracea induced apoptosis and down-regulated cell survival and antioxidant markers in MCF-7 cells [34]. The inhibitory effect of lycopene on the growth of cancer cells depends on the level of cyclin D1, which cuts off the transition of MCF-7 cells from the G1 to the S phase [35]. In addition, carotenoid lutein induces the mitochondrial apoptosis pathway in breast cancer cells through ROS-mediated mechanisms [36]. Lycopene-rich and β-carotene extracts from guava fruit exert anti-proliferative effects on MDA-MB-435 and MCF-7 breast cancer cell lines [37].

Regarding the low toxicity of carotenoids and their potential anticancer effects in reducing tumor growth, numerous studies are needed to further illustrate the possible processes and molecular mechanisms of carotenoids’ anticancer effects. Several studies have focused on linking individual carotenoids to different types of cancer risk assessment. However, there are few reports on the anticancer activity and molecular mechanisms of carotenoid extracts from various citrus resources. In our previous studies [9], we found that citrus pulp has abundant and diverse carotenoids, and also exhibited higher anti-proliferative activity against cancer cells. However, the effects of different citrus carotenoid extracts on the proliferation, cell cycle, and apoptosis of triple-negative breast cancer cells and their possible mechanisms are still unclear. In this study, we aim to investigate the effects of citrus pulp carotenoid extracts on the proliferation, production of reactive oxygen species (ROS), intracellular redox state, cell cycle, and apoptosis of MDA-MB-231 cells (a triple-negative breast cancer cell). In addition, we examined the expression of genes related to mitochondrial endogenous signaling pathways that cause apoptosis.

## 2. Materials and Methods

### 2.1. Plant Materials

Based on previous studies, we selected five citrus varieties with large cultivation scales. The five cultivars included three mandarins, ‘ZaoMiPengGan’, ‘DongFangHong’, and ‘NanFengMiJu’; one orange, ‘ZaoHongQiCheng’; and one grapefruit, ‘XiYou’ (Table 1). The citrus fruits from these cultivars were fully ripe when we collected them. The citrus pulp and peel were separated, then frozen in liquid nitrogen, and pulverized using a multi-functional grinder. The citrus powder was used for subsequent analyses.

### 2.2. Reagents

The PBS, 2,7-dichlorodihydro-fluorescein diacetate (DCFH-DA) probe, and cell cycle analysis kit were purchased from biosharp (Hefei, China). The dimethyl sulfoxide (DMSO), penicillin and streptomycin (P/S), microplate reader assay kits (superoxide dismutase (SOD), catalase (CAT), peroxidase (POD), glutathione reductase (GR), malondialdehyde (MDA) and glutathione (GSH)), and bicinchoninic acid (BCA) protein assay kit were purchased from Solarbio Life Sciences (Beijing, China). The mitochondrial membrane potential detection kit was also obtained from Solarbio at a concentration of 10 µM. The annexin V conjugated with fluorescein isothiocyanate/propidium iodide (ANNEXIN V-FITC/PI) apoptosis kit was sourced from Elabscience (Wuhan, China). The cytotoxicity kit CCK8 was obtained from GLPBIO (Shanghai, China). BeyoClick™ EdU Cell Proliferation Kit was sourced from Beyotime (Shanghai, China).

### 2.3. Carotenoids Extraction

The solvent and method of extracting carotenoids from peels or pulps were adapted from the procedures described by Kaijie Zhu and Rodriguez-Amaya et al. [38,39], with slight modifications. The citrus pulp powder was poured into a 50 mL centrifuge tube, and then 25 mL of hexane/acetone/ethanol (2:1:1) with 0.1% butylated hydroxytoluene (BHT) was added for 30 min under ultrasonic conditions. The supernatant was transferred to a new tube, and this was repeated three times. The combined organic layer was evaporated by a rotary vacuum evaporator at 30 °C, and the concentrated extracts were dried with nitrogen and then stored at −20 °C.

### 2.4. Cell Culture

The human breast cancer MDA-MB-231 cells were purchased from Procell Life Science & Technology Co., Ltd. (Wuhan, China). MDA-MB-231 cells were cultured in DMEM containing 10% FBS and 1% P/S (Procell, Wuhan, China). Cells were incubated in a 5% CO_2_ incubator at 37 °C. Cells were incubated for 24 h before treatment with the carotenoid extracts.

### 2.5. Cell Cytotoxicity Assays

Referring to Wen Xia et al.’s method [2], the MDA-MB-231 cells were cultured at the exponential proliferation phase and seeded at 1 × 10^4^ cells per well into 96-well plates. The cells were incubated at 37 °C in a 5% CO_2_ incubator. After 24 h of incubation, the medium was replaced with fresh medium with different carotenoid extract concentrations (12.5~200 µg/mL) for the treatments and fresh medium with 0.5% DMSO for the control. The cells were incubated with the carotenoid extracts for 48 h, after which the existing medium was discarded, and the cells were washed with PBS. Then, the cytotoxicity was determined using CCK8 kits, and the percentage of cell loss under control and different treatments was calculated. We used the software OriginPro 2021 to fit the absorbance value measured at 450 nm to obtain the fitting curve and the IC50. Experiments were performed three times.

### 2.6. Cell Proliferation Assays

To evaluate the anti-proliferation effect of citrus pulp carotenoid extracts in a dose-dependent manner, we used a BeyoClick™ EdU Cell Proliferation Kit (Alexa Fluor 555, Beyotime, Shanghai, China) for analysis. The experiment was carried out according to the manufacturer’s protocol. MDA-MB-231 cells were treated with different concentrations of carotenoid extracts, incubated with 10 µM 5-ethynyl-2′-deoxyuridine (EDU) solution for 2 h at 37 °C, and then fixed with 4% paraformaldehyde for 15 min. After washing with PBS three times, the cells were incubated with Hoechst 33342 for 10 min. Finally, the cells were analyzed and imaged using a fluorescence microscope (Olympus U-HGLGPS, Shanghai, China).

### 2.7. Cell Apoptosis and Cycle Analysis

MDA-MB-231 cells (3 × 10^5^ cells/well) were seeded into 6-well plates and incubated for 24 h. Then, the cells were treated with different carotenoid extracts for 48 h. The cells were harvested and washed twice with cold 1 × PBS and then resuspended in 1 × Annexin V binding buffer. Subsequently, 2.5 µL of Annexin V-FITC and 2.5 µL of PI were added and the cells were stained for 15 min in the dark for apoptosis analysis. Cells used for cell cycle measurement were fixed in 70% ethanol at 4 °C overnight. Then, 0.5 mL of PI was added to stain them at 37 °C for 30 min. The distribution of the cell cycle and cell apoptosis were determined by flow cytometry (BD, PerkinElmer, Shelton, CT, USA).

### 2.8. Measurement of Mitochondrial Membrane Potential (MMP)

The mitochondrial membrane potential was evaluated by a JC-1 assay kit. MDA-MB-231 cells were cultured for 24 h and treated with carotenoid extracts for 48 h in 6-well plates in 5% CO_2_ at 37 °C. After treatment, the cells were incubated with JC-1 solution for 20 min at 37 °C. Then, the cells were washed twice with JC-1 buffer solution, the fluorescence images were detected by a fluorescence microscope (Olympus U-HGLGPS, Shanghai, China), and the fluorescence intensity was detected by a multifunctional microplate reader.

### 2.9. Measurement of ROS Level

To detect the intracellular ROS level, MDA-MB-231 cells were seeded in 96-well plates and cultured overnight in 5% CO_2_ at 37 °C. Then, the cells were treated with carotenoid extracts for 48 h. Following treatments, the cells were incubated with serum-free DMEM containing a DCFH-DA probe (10 µM) for 30 min at 37 °C in the dark. Then, the cells were washed twice with serum-free DMEM, and the fluorescence intensity of the cells was imaged using cellSens dimension software under a fluorescence microscope (Olympus U-HGLGPS, China).

### 2.10. Oxidative Factor Detection

Based on the principle of colorimetry, the enzyme activities of SOD and POD were determined according to the instructions of the kits, and the absorbance values were read at 560 nm and 470 nm using a microplate reader. Based on the determination of GR activity by ultraviolet colorimetry, GR can catalyze NADPH to reduce oxidized glutathione (GSSG) to regenerate glutathione (GSH), and NADPH is dehydrogenated to NADP+. The rate of NADPH dehydrogenation was determined by measuring the decrease in absorbance at 340 nm, and the GR activity was calculated. The CAT enzyme activity was determined by ultraviolet colorimetry. CAT decomposes H_2_O_2_, causing the absorbance of the reaction solution at 240nm to decrease with the reaction time. The CAT activity can be calculated based on the change rate of the absorbance. The levels of malondialdehyde (MDA) and glutathione (GSH) were separately detected by assay kits, and the absorbance values were read using a microplate reader. Protein content was measured using a BCA kit.

### 2.11. Real-Time PCR Determination

Total RNA was extracted using the total RNA separation kit according to the manufacturer’s instructions (TIANGEN, Beijing, China). The mRNA was reverse transcribed into cDNA using a cDNA synthesis kit (Vazyme, Nanjing, China). After cDNA synthesis, quantitative real-time PCR (qRT-PCR) was performed using Bio-Rad CFX 96 with SYBR Green Master Mix (Vazyme, Nanjing, China). The *GAPDH* gene was used as the internal reference gene. The primer sequence of the gene is shown in Supplementary Table S1. All real-time PCR experiments were repeated three times, and the values were expressed as mean data.

### 2.12. Statistical Analysis

All experiments were repeated in triplicate. The data were expressed as mean ± SD. The cytotoxicity data were analyzed and fitted using Origin 2021. The differences among multiple samples were tested by ANOVA using Tukey’s multiple comparison tests in GraphPad prism 8.0.2 software. *p*-values of <0.05 or <0.01 were considered significant.

## 3. Results

### 3.1. Carotenoid Extracts Inhibited Viability in MDA-MB-231 Cells

**Figure 1:**
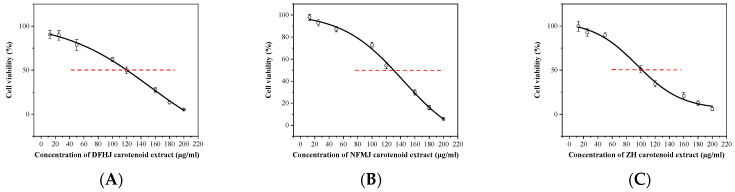

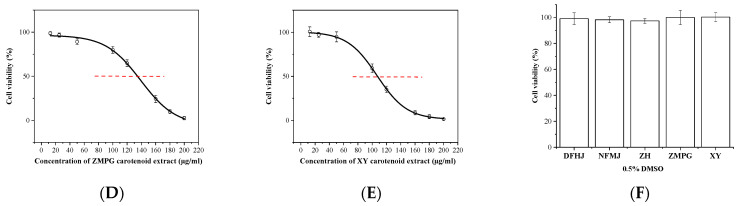
Effect of different citrus pulp carotenoid extracts on the viability of MDA-MB-231 cells. (**A**–**E**) MDA-MB-231 cells were treated with various concentrations of DFHJ, NFMJ, ZH, ZMPG, and XY carotenoid extracts for 48 h. (**F**) The control was treated with 0.5% DMSO. Cell viability was measured by CCK8 kit. The data represent the mean ± SD.

Our previous study detected and identified nine carotenoid compounds in citrus pulp through HPLC (Table 2), and found that citrus fruits mainly contain β-cryptoxanthin, zeaxanthin, lutein, β-carotene, 9-cis-violaxanthin, and violaxanthin. Notably, XY mainly contains β-carotene and lycopene [9]. Citrus contains different types and contents of carotenoids due to its different varieties. Our previous research found that carotenoid extracts from citrus pulp had anticancer effects. To quantify the cytotoxicity of carotenoid extracts on MDA-MB-231 breast cancer cells, we analyzed the inhibitory effect of five carotenoid extracts on cell viability. The cells were incubated with different concentrations of carotenoid extracts (0, 12.5, 25, 50, 100, 120, 160, 180, and 200 µg/mL) for 48 h, and a CCK8 assay was used to evaluate cell viability. Carotenoid extracts significantly suppressed the MDA-MB-231 cell viability in a dose-dependent manner, and the 48 h IC50 in MDA-MB-231 cells was different in carotenoid extracts from different citrus species (Figure 1). The IC50 values of DFHJ, NFMJ, ZH, ZMPG, and XY carotenoid extracts were 152.46 µg/mL, 143.09 µg/mL, 94.89 µg/mL, 138.16 µg/mL, and 106.76 µg/mL, respectively (Table 3). For MDA-MB-231 cells, treatment with 0.5% DMSO did not affect cell viability (Figure 1F). Considering the IC50 values of different citrus pulp carotenoid extracts in MDA-MB-231 cells, we found that among the five carotenoid extracts, the XY and ZH carotenoid extracts exhibited higher inhibitory effects on cell growth. Therefore, the XY and ZH carotenoid extracts were chosen for the subsequent experiments, and the experimental concentrations were 50 µg/mL and 100 µg/mL. Among them, the concentration of XY1 and ZH1 was 50 µg/mL, while the concentration of XY2 and ZH2 was 100 µg/mL.

### 3.2. Effect of Carotenoid Extracts on Proliferation in MDA-MB-231 Cells

**Figure 2:**
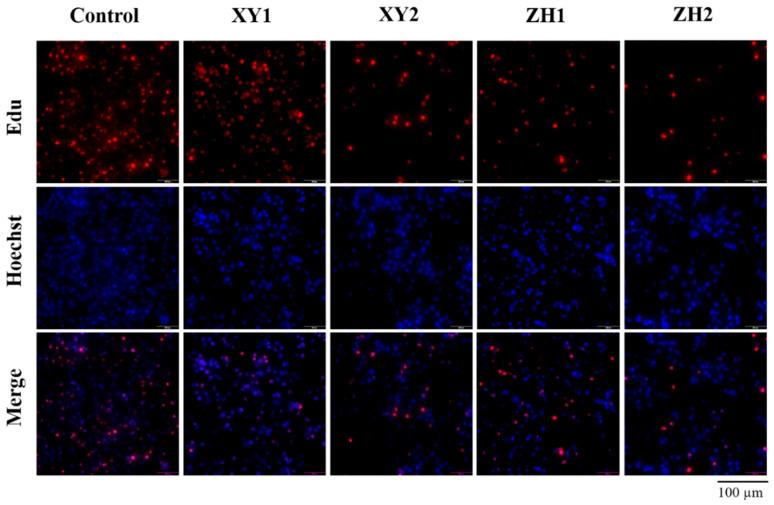
Effect of carotenoid extracts of XY and ZH on proliferation and migration in MDA-MB-231 cells. The cells were treated with XY1 (50 µg/mL), XY2 (100 µg/mL), ZH1(50 µg/mL), and ZH2 (100 µg/mL); then, BeyoClick™ EdU-555 kit and Hoechst 33342 were used to analyze cell proliferation. Mean ± SD. Control was treated with 0.5% DMSO.

To investigate the impact of carotenoid extracts on the proliferation of MDA-MB-231 cells, we performed an EDU staining assay to confirm the inhibitory effect of ZH and XY on cell proliferation. The EDU proliferation assay is based on the incorporation of thymidine analog EdU (5-ethynyl-2′-deoxyuridine) during DNA synthesis, and EdU is labeled by subsequent click reactions to detect cell proliferation. As presented in Figure 2, we observed that the proliferation of cells represented by red fluorescence was inhibited in XY and ZH compared with the control. With the increase in XY and ZH concentration, MDA-MB-231 cell proliferation was significantly inhibited in a concentration-dependent manner. Moreover, compared with XY1 and ZH1, XY2 and ZH2 had the strongest inhibitory effect on cell proliferation.

### 3.3. Effect of Carotenoid Extracts on Cell Cycle in MDA-MB-231 Cells

**Figure 3:**
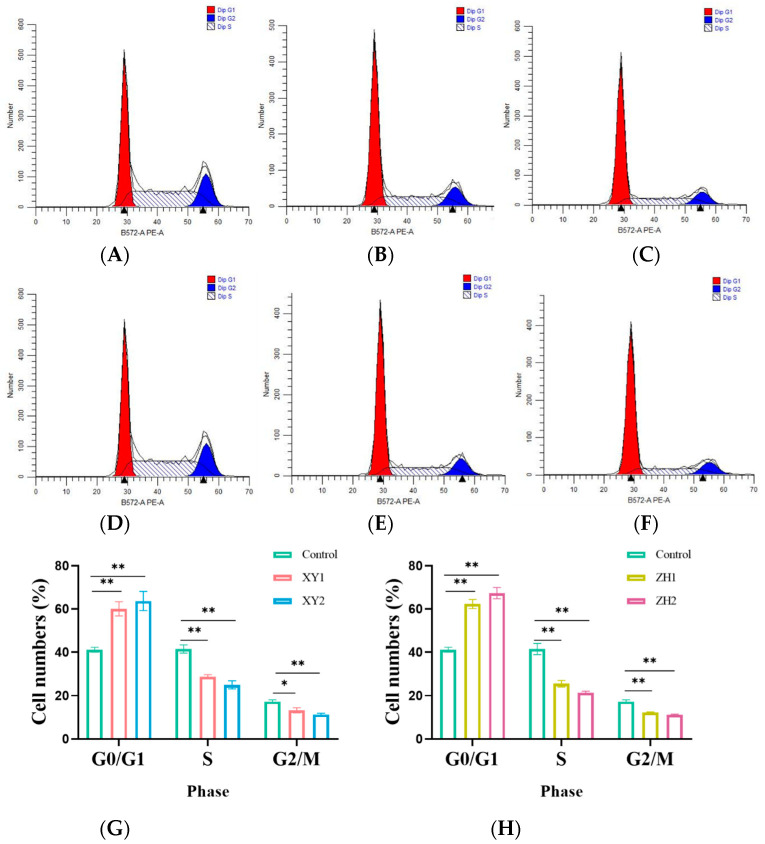
Carotenoid extracts of XY and ZH induce cell cycle arrest in MDA-MB-231 cells. (**A**–**F**) Cell cycle distributions detected by flow cytometry; (**A**–**F**) represents control, XY1, XY2, control, ZH1, and ZH2. (**G**,**H**) The percentage of cells in different phases. XY1 and ZH1 are 50 µg/mL treatment concentrations; ZH1 and ZH2 are 100 µg/mL treatment concentrations. The data are presented as mean ± SD. * *p* < 0.05, ** *p* < 0.01.

To examine the potential role of XY and ZH in cell growth inhibition, cell cycle analysis was implemented. As shown in Figure 3, MDA-MB-231 cells were treated with XY and ZH, respectively, for 48 h, and the cell distribution of PI staining was analyzed by flow cytometry. The results showed that G0/G1 phase cells increased in a dose-dependent manner after being exposed to XY and ZH for 48 h. Correspondingly, the cells were significantly reduced in the S phase and G2/M phase, suggesting that XY and ZH treatment can significantly block the G0/G1 phase of tumor cells. More obviously, the percentage of cells in G0/G1 increased by 21.17% and 26.20% in the XY2 and ZH2 treatment groups, respectively (Figure 3G,H). Furthermore, the percentage of cells in the S phase also decreased rapidly, decreasing by 16.03% and 20.12% in the XY2 and ZH2 treatments at 100µg/mL, respectively. Thus, these results strongly indicate that XY and ZH induced G0/G1 arrest in MDA-MB-231 cells.

### 3.4. Effect of Carotenoid Extracts on Cell Apoptosis in MDA-MB-231 Cells

**Figure 4:**
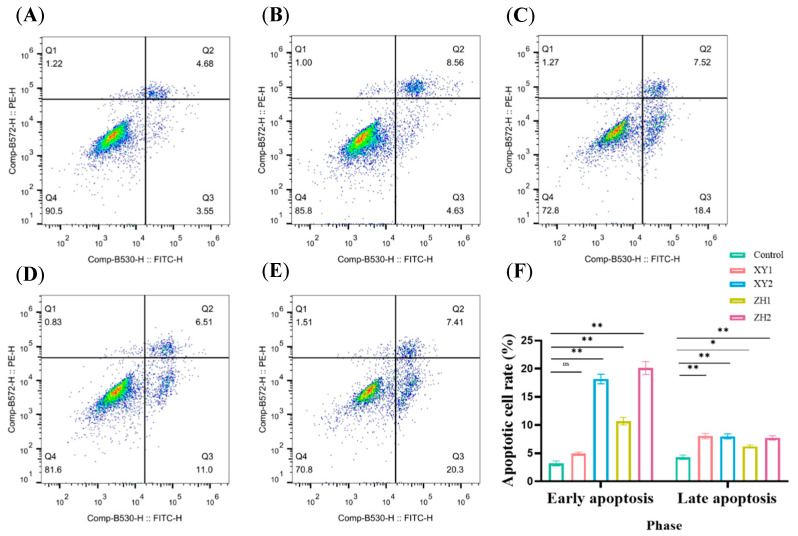
Carotenoid extracts of XY and ZH induce cell apoptosis in MDA-MB-231 cells. (**A**–**E**) Apoptotic cells were assessed by flow cytometry after Annexin V-FITC and PI staining. Q1 represents necrotic cells; Q2 represents late apoptotic cells; Q3 represents early apoptotic cells; Q4 represents viable cells. A-E represents control, XY1, XY2, ZH1, and ZH2. (**F**) Apoptotic cell rate in early and late phases. XY1 and ZH1 are 50 µg/mL treatment concentrations; ZH1 and ZH2 are 100 µg/mL treatment concentrations. The data are presented as mean ± SD. * *p* < 0.05, ** *p* < 0.01.

To further evaluate whether the growth inhibition induced by XY and ZH is dependent on apoptosis, we used flow cytometry to analyze the apoptotic effects of XY and ZH on MDA-MB-231 cells. As shown in Figure 4, cells were treated with XY and ZH for 48 h, and the percentages of living cells, early apoptotic cells, and late apoptotic cells were determined. Compared with the control, XY and ZH significantly increased cell apoptosis in a concentration-dependent manner, especially XY2 and ZH2. Treatment with XY2 and ZH2 in MDA-MB-231 cells significantly increased the early apoptotic cells by 14.98% and 16.92%, and the late apoptotic cells by 3.69% and 3.45%, suggesting that XY and ZH mainly induced early apoptosis (Figure 4F). XY1 and ZH1 could also promote the early apoptosis of cells, and the proportion of apoptosis was 4.92% and 10.69%, respectively. The results showed that XY and ZH can induce apoptosis in MDA-MB-231 cells.

### 3.5. Carotenoid Extracts Cause ROS Accumulation and Decrease Mitochondrial Membrane Potential (MMP) 

**Figure 5:**
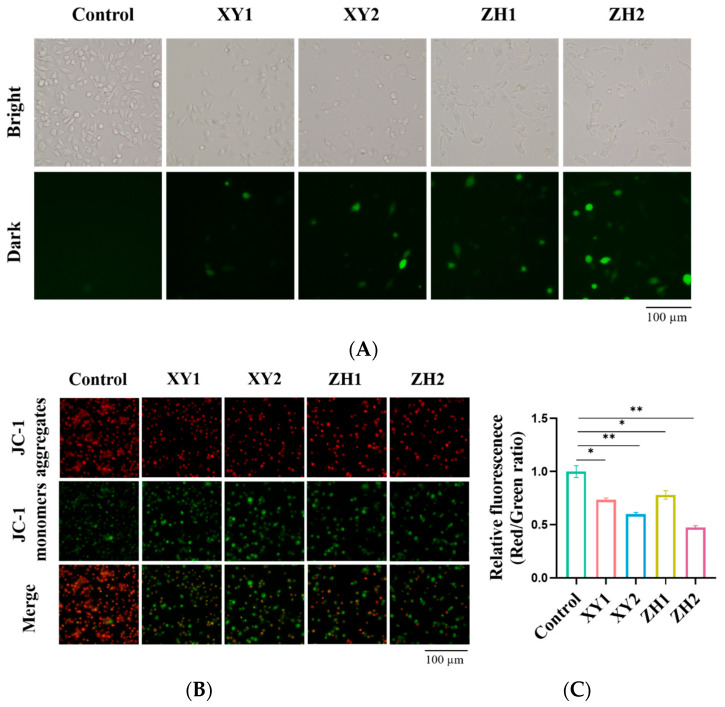
Carotenoid extracts of XY and ZH cause ROS accumulation and MMP changes in MDA-MB-231 cells. (**A**) Assessment of intracellular ROS level uses a DCFH-DA probe after treatment with XY and ZH. (**B**) Fluorescence images of MDA-MB-231 incubated with JC-1 after treatment with XY and ZH. (**C**) MMP quantified by measuring green fluorescence intensity and red fluorescence intensity. Mean ± SD. * *p* < 0.05, ** *p* < 0.01. XY1 = 50 µg/mL, XY2 = 100 µg/mL, ZH1 = 50 µg/mL, ZH2 = 100 µg/mL.

An increase in ROS levels is a product of oxidative stress in cells, and their excessive production can destroy biological macromolecules and induce apoptosis in cells [40]. To detect the effect of XY and ZH on the level of ROS in MDA-MB-231 cells, a DCFH-DA probe was incubated with cells and imaged by fluorescence microscopy. We observed that both XY and ZH appreciably increased intracellular ROS levels in a dose-dependent manner in MDA-MB-231 cells (Figure 5A). As compared to ROS levels in the XY1- and ZH1-treated groups, the XY2 and ZH2 groups exhibited the strongest fluorescence intensity, indicating the highest ROS level in cells. Thus, the results indicated that XY and ZH could increase the accumulation of ROS in breast cancer cells and cause oxidative stress.

To prove whether XY and ZH can decrease MMP, we used a JC-1 probe to analyze the alterations in the MMP of MDA-MB-231 cells. A significant difference was observed between the green fluorescence of JC-1 monomers and the red fluorescence of JC-1 aggregate. As shown in Figure 5B,C, the cells treated with XY and ZH showed an increase in green fluorescence intensity and a decrease in red fluorescence intensity in a concentration-dependent manner after 48 h. At 100 µg/mL, XY2 and ZH2 significantly enhanced the green fluorescence signal. The red/green ratio indicated the loss of MMP, which declined to 0.74, 0.60, 0.78, and 0.47 when treated with XY1, XY2, ZH1, and ZH2 concentrations, respectively, compared to the control. Hence, XY and ZH have the potential to reduce MMP and affect mitochondrial function in breast cancer cells.

### 3.6. Carotenoid Extract Treatment Enhanced the MDA Level and Reduced the GSH Level in MDA-MB-231 Cells

**Figure 6:**
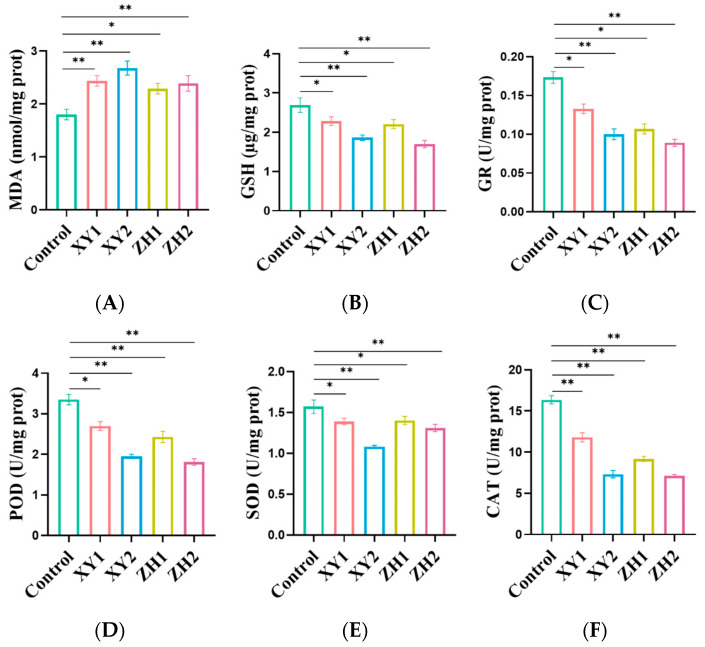
Effects of XY and ZH carotenoid extracts on the levels of MDA (**A**), GSH (**B**), GR (**C**), POD (**D**), SOD (**E**), and CAT (**F**) in MDA-MB-231 cells. Mean ± SD. * *p* < 0.05, ** *p* < 0.01. XY1 = 50 µg/mL, XY2 = 100 µg/mL, ZH1 = 50 µg/mL, ZH2 = 100 µg/mL.

Lipid peroxidative damage to cell membranes is one of the main consequences of excessive ROS production, among which the production of the secondary product malondialdehyde (MDA) is one of the markers of oxidative damage [41]. Compared to the control group, the results showed that the level of MDA significantly increased in a dose-dependent manner after 48 h of incubation with different concentrations of XY and ZH (Figure 6A). XY1 and XY2 increased MDA levels by 35.37% and 48.89%, respectively. Similarly, increasing the concentration of ZH-treated MDA-MB-231 cells also resulted in a significant increase in MDA levels, with respective increases of 27.22% and 32.78%. These findings demonstrate that both XY and ZH have the ability to significantly elevate MDA levels, leading to oxidative damage in cells.

According to Figure 6B, the levels of GSH of the MDA-MB-231 cells treated with XY1 and XY2 decreased by 15.12% and 30.86%, respectively, compared with the control. The GSH content in MDA-MB-231 treated with ZH1 and ZH2 was lower than that of the control, indicating that ZH had a significant effect on reducing GSH levels. The results showed that XY and ZH treatment reduced GSH levels, resulting in decreased levels of scavenging ROS, and thereby increasing oxidative stress in cells.

### 3.7. Antioxidant Enzyme Activity of MDA-MB-231 Cells

GR, SOD, POD, and CAT, as the main contributors to the antioxidant enzyme system, play an important role in maintaining cell redox homeostasis and preventing oxidative damage [42]. As shown in Figure 6C, XY and ZH treatment of MDA-MB-231 cells for 48 h significantly reduced GR levels in a dose-dependent manner. Compared to the control, the administration of XY2 and ZH2 significantly decreased the levels of GR, which declined by 42.31% and 48.65%, respectively. Moreover, XY1 and ZH1 also reduced the activity of GR, which decreased by 23.27% and 38.33%, respectively, as compared with the control group. To summarize, XY and ZH significantly inhibited the activity of GR in MDA-MB-231 cells.

In Figure 6D, it is shown that both XY and ZH significantly decreased the activity of POD compared to the control group. This suggests that there is a dose-dependent relationship between the activity of POD and the concentrations of XY and ZH. In the case of XY, the POD activity in MDA-MB-231 cells was reduced by 19.50% and 41.79% at concentrations of 50 and 100 µg/mL, respectively. Similarly, for ZH, the POD activity was reduced by 27.46% and 45.87% at concentrations of 50 and 100 µg/mL, respectively. POD activity was greatly affected by XY and ZH, and the enzyme activity was significantly inhibited.

Figure 6E shows that the enzymatic activity of SOD decreased with increasing concentrations of XY and ZH. In comparison, the activity of SOD was significantly reduced by XY2 and ZH2, by 31.21% and 16.56%, respectively. XY1 and ZH1 treatment reduced the levels of SOD by 11.46% and 10.83%, respectively, compared with the control group. It can be seen that in MDA-MB-231 cells, both ZH and XY had a significant inhibitory effect on POD enzymatic activity.

As shown in Figure 6F, the CAT activity of XY1 and XY2 decreased by 27.86% and 55.19% at 50 and 100 µg/mL, respectively, which was significantly lower than that of the control group. For ZH1 and ZH2, the activity of CAT decreased by 43.86% and 56.35%, respectively, in a concentration-dependent manner when the cells were treated with 50 and 100 µg/mL. The above results show that CAT activity was significantly inhibited with the increase in XY and ZH treatment concentration.

### 3.8. Analysis of Gene Expression Changes in Antioxidative-Capacity-Related Genes in MDA-MB-231 Cells after Treatment with Carotenoid Extracts

**Figure 7:**
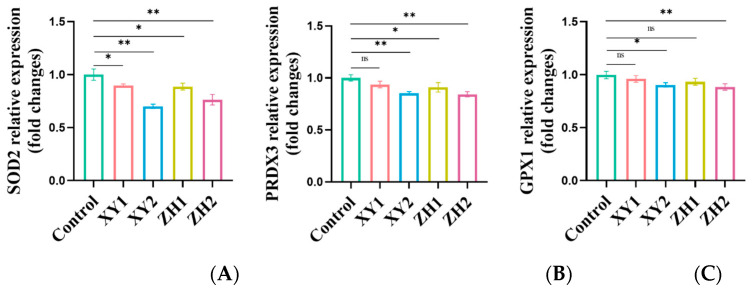
Effects of XY and ZH carotenoid extracts on antioxidant-related genes. (**A**) SOD2, (**B**) PRDX3, (**C**) GPX1. Mean ± SD. * *p* < 0.05, ** *p* < 0.01. XY1 = 50 µg/mL, XY2 = 100 µg/mL, ZH1 = 50 µg/mL, ZH2 = 100 µg/mL; ns represents no statistically significant difference.

To further elucidate carotenoid-extract-induced changes in cell redox state, the gene expression of superoxide dismutase 2 (*SOD2*), peroxiredoxin 3 (*PRDX3*), and glutathione peroxidase 1 (*GPX1*) was detected by qRT-PCR. From Figure 7, the *SOD2* mRNA expression level with XY treatment was downregulated by 0.90-fold and 0.7-fold at 50 and 100 µg/mL, respectively, and the *SOD2* mRNA expression level with ZH treatment was downregulated by 0.89-fold and 0.76-fold at 50 and 100 µg/mL, respectively. MDA-MB-231 cells exposed to XY2 and ZH2 showed a significant reduction in *PRDX3* at 100 µg/mL with 0.86-fold and 0.84-fold, respectively, compared to the control. An elevation of *PRDX3* at XY1 to 0.93-fold was found to be non-significant, while at ZH1 to 0.91-fold it was found to be significant. In the case of *GPX1*, there was a significant decrease in *GPX1* at XY1 and XY2 to 0.96-fold and 0.90-fold, and at ZH1 and ZH2 to 0.93-fold and 0.88-fold.

### 3.9. Analysis of Gene Expression Changes in Antioxidative-Capacity-Related Genes in MDA-MB-231 Cells after Treatment with Carotenoid Extracts

**Figure 8:**
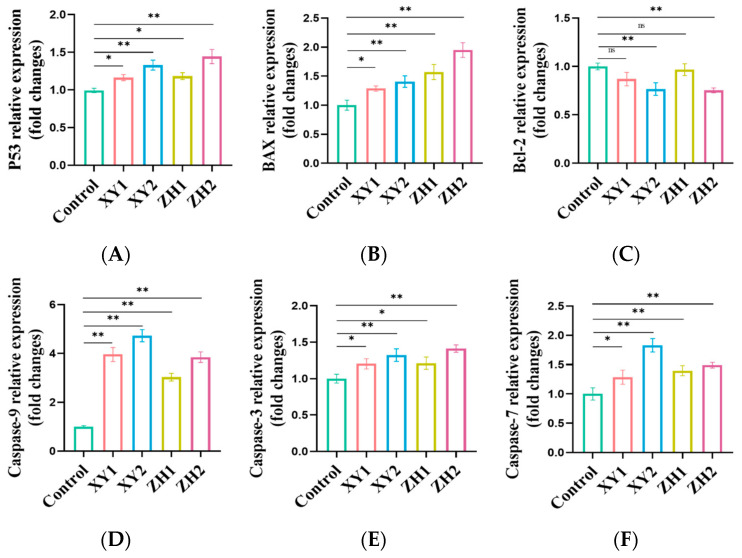
The relative mRNA expression of apoptosis-related genes in MDA-MB-231 cells. (**A**) P53, (**B**) BAX, (**C**) Bcl-2, (**D**) caspase-9, (**E**) caspase-3, (**F**) caspase-7. Mean ± SD. * *p* < 0.05. ** *p* < 0.01. XY1 = 50 µg/mL, XY2 = 100 µg/mL, ZH1 = 50 µg/mL, ZH2 = 100 µg/mL. ns represents no statistically significant difference.

To further evaluate the effect of carotenoid extracts on apoptosis, the expression of apoptosis-related genes was detected by qRT-PCR. In our study, the up-regulation of *P53* mRNA expression in a concentration-dependent manner at different concentrations of XY and ZH was consistent with apoptosis and cell cycle arrest in flow cytometry experiments. After treatment with 50 µg/mL of XY and ZH, the mRNA expression of *P53* was up-regulated by 1.16-fold and 1.18-fold. The *P53* gene was significantly upregulated with XY2, and ZH2 concentrations to 1.32- and 1.45-fold, respectively, compared to control (Figure 8A). The results showed that the expression levels of pro-apoptotic gene *BAX* and anti-apoptotic gene *Bcl-2* in MDA-MB-231 cells treated with XY and ZH were significantly changed (Figure 8B,C). The expression of the *Bcl-2* gene was significantly downregulated by 0.87-, 0.76-, 0.96-, and 0.75-fold in the treated cells after treatment with XY1, XY2, ZH1, and ZH2, compared to the control. However, the *BAX* gene was significantly upregulated to 1.29-fold, 1.34-fold, 1.57-fold, and 1.59-fold, respectively, at the XY1, XY2, ZH1, and ZH2 concentrations compared to the control. Meanwhile, we also observed a significant increase in the *BAX*/*Bcl-2* ratio with increasing concentrations of XY and ZH treatments. A significant increase in the *BAX*/*Bcl-2* ratio indicated apoptosis induction through activating caspase cascades, such as *caspase-3*, *caspase-7*, and *caspase-9*. In Figure 8D, compared with the control, *caspase-9* gene expression was increased in a concentration-dependent manner after XY1, XY2, ZH1, and ZH2 treatment, by 3.96-, 4.72-, 3.03-, and 3.85-fold, respectively. *Caspase-3* was up-regulated to 1.20-fold and 1.22-fold at the XY1 and ZH1 concentrations. The expression of the *caspase-3* gene was significantly up-regulated by 1.32-fold and 1.41-fold after XY2 and ZH2 treatment, respectively (Figure 8E). Similarly, the expression of *caspase-7* was also found to be increased by 1.29-, 1.83-, 1.39-, and 1.49-fold at the XY1, XY2, ZH1, and ZH2 concentrations, respectively, in a concentration-dependent manner (Figure 8F).

## 4. Discussion

Breast cancer is a complex and destructive malignant tumor that affects women around the world, regardless of the country’s development. We need to develop a more effective and non-toxic new strategy to inhibit the growth of cancer cells and fight tumors. Therefore, various bioactive compounds from natural products have received extensive attention as potential cancer therapies [6,43]. Carotenoids, which are known for their powerful antioxidant/pro-oxidant properties, have shown promise in cancer prevention/inhibiting [20]. Citrus fruits, which are a rich source of carotenoids, contain the highest carotenoid content among all fruits [44]. A previous study demonstrated that XY and ZH have abundant carotenoid content and types, which exert significant anti-proliferation, pro-apoptosis, and oxidative stress effects on MCF-7 breast cancer cells [9]. In view of the fact that the therapeutic targets and optimal treatments for triple-negative breast cancer have yet to be determined, it may be a good choice to choose natural, non-toxic carotenoid extracts as a multi-process, multi-pathway, and multi-target treatment modality.

Growing evidence suggests that the potential anticancer mechanisms of carotenoid extracts incorporate antiproliferation, cell cycle arrest, induction of oxidative stress, and pro-apoptosis processes by regulating cell signal transduction pathways [45]. Therefore, the purpose of our study was to evaluate the effects of carotenoid extracts derived from various citrus species in MDA-MB-231 human breast cancer cells, including cellular redox state (ROS generation and antioxidant enzyme activity) and apoptosis via a mitochondrial-mediated pathway. The carotenoid extracts were found to have concentration-dependent effects on cell growth, as determined by the CCK8 assay. The order of observed cytotoxicity, based on IC50 values, was ZH > XY > ZMPG > NFMJ > DFHJ. Among these carotenoid extracts, treatment with ZH and XY significantly inhibited the growth of MDA-MB-231 cells, with higher concentrations showing a greater toxic effect on the cells. Consistent with the results of cell viability, treatment with XY and ZH significantly inhibited the proliferation of MDA-MB-231 cells and reduced the growth of cancer cells in a concentration-dependent manner. Studies have shown that the combination of carotenoids at equimolar concentrations can inhibit the growth of cancer cells more effectively than individual carotenoids [32]. Another study also demonstrated that carotenoid extracts effectively inhibited the activity of MCF-7 human breast cancer cells and suppressed tumor growth in female BALB/c nude mice [46]. Russo M et al. proposed that the carotenoid mixture exhibits a stronger inhibitory effect on MCF-7 cell proliferation, which may be attributed to its synergistic effect [47]. Similarly, Linnewiel-Hermon et al. also believed that the synergistic inhibition of the growth of prostate cancer cells and breast cancer cells was obvious when various carotenoids were combined at low concentrations [48]. Although these studies were limited to a single cell line, this suggests that the synergistic activity of carotenoid mixtures on cancer cell proliferation is also confirmed by other reports and that these carotenoid extracts or mixtures can be tested in combination with traditional chemotherapy drugs [49]. In the present study, we further demonstrated that carotenoid extracts have anti-proliferation effects on MDA-MB-231 breast cancer cells.

ROS include superoxide radical (O_2_^•−^), hydroxyl radical (^•^OH), hydrogen peroxide (H_2_O_2_), and peroxyl radical, which play an important role in cancer proliferation, apoptosis, and regulating cell homeostasis [50,51]. Due to the sensitivity of cancer cells to ROS, increased ROS levels or decreased ROS scavenging capacity may cause cancer cells to exceed the breaking point in terms of DNA damage, lipid peroxidation, and protein oxidation, leading to programmed cell death [40,52]. Recently, this biochemical basis has attracted attention as a new therapeutic approach, particularly for oxidative stress in cancer cells induced by exogenous ROS generators that disrupt their own antioxidant systems and trigger oxidative-stress-induced apoptosis in cancer cells [53]. In this study, we found that carotenoid extracts enhanced the accumulation of ROS in MDA-MB-231 cells, which significantly affected cellular redox homeostasis and even induced apoptosis through oxidative stress in cells. Lutein-treated breast cancer cell lines (MCF-7 and MDA-MB-468) and HeLa cells show enhanced accumulation of ROS, leading to reduced cell viability and triggering apoptosis [54,55]. Previous studies have shown that lycopene and lycopene oxidation products increase ROS levels and induce apoptosis in breast cancer cells [56]. Oxidative stress occurs when ROS production increases while non-enzymatic defense system (GSH) and antioxidant enzyme defense system levels decrease [57]. As part of maintaining intracellular redox homeostasis, GSH plays a vital role in scavenging ROS, and their reduced level mediates breast cancer cell apoptosis [58]. We observed that the GSH levels in MDA-MB-231 cells decreased in a dose-dependent manner. In addition, MDA, as a marker of oxidative stress [41], was significantly increased in carotenoid-extract-treated cells. The consumption of GSH and the increase in MDA levels reflect lipid peroxidation in mitochondria, indicating an increase in oxidative stress [59]. The antioxidant enzyme system is essential for maintaining intracellular redox homeostasis and combating oxidative stress by neutralizing free radicals [60]. As an antioxidant enzyme that directly catalyzes the conversion of hydrogen peroxide [61], the activity of CAT decreased after treatment with carotenoid extracts, indicating a reduction in ROS scavenging ability. SOD serves as the first line of defense against ROS, catalyzing the conversion of superoxide radicals into hydrogen peroxide (H_2_O_2_), which is catalyzed by CAT to produce H_2_O and O_2_, thereby eliminating ROS and protecting cells from H_2_O_2_ damage. Moreover, the regeneration of GSH depends on the participation of GR, which is also an important process for cells to prevent oxidation [62]. The increase in membrane lipid peroxidation product MDA level, the consumption of GSH, and the decrease in antioxidant enzyme activity (SOD, POD, CAT, GR) suggested that the treatment of carotenoid extracts disrupted the cellular antioxidant reduction system, resulting in oxidative stress in MDA-MB-231 cells. Studies have found that β-carotene, lutein, and astaxanthin, either individually or in an equal concentration mixture of three carotenoids, have pro-oxidative effects in MCF-7 and MDA-MB-231 cells, and subsequently, ROS and malondialdehyde (MDA) levels increase, and glutathione (GSH) levels are reduced [32]. Arathi, B.P. et al. have shown that lycopene and its oxidation products inhibit the viability of cancer cells while promoting the increase in ROS and MDA levels and the decrease in GSH levels [63]. Compared with normal cells, tumor cells have lower levels of antioxidant enzymes, such as SOD, CAT, GPX, and GR, which hinder the detoxification process of free radicals and maintain high intracellular ROS levels [64]. Further, the study found that carotenoid extract treatment also down-regulated the antioxidant-related gene expression of SOD2, PRDX3, and GPX1. Taken together, the anti-cancer effect of carotenoid extracts may be due to the excessive production of pro-oxidant factors such as ROS and MDA, as well as the reduced activity of the antioxidant enzyme activity system, including GR, POD, SOD, GSH, and CAT.

During normal cell growth, there exists an internal dynamic equilibrium between cell proliferation and apoptosis. The occurrence of tumors is primarily due to uncontrolled cell growth, which is characterized by disordered cell proliferation and impaired apoptosis [65]. Within cancer cells, the pro-oxidant activity of carotenoids causes oxidative injury, which hinders the proliferation of cancer cells because oxidative stress restricts the progression and metastasis in cancer cells [26]. Cell cycle regulation is often closely related to biological processes such as cell proliferation and apoptosis. Our study found that MDA-MB-231 cells treated with carotenoid extracts were arrested in the G0/G1 phase, which was consistent with the previous cell anti-proliferation study, indicating that carotenoid extracts have an inhibitory effect on human breast cell lines. This is consistent with a previous study in which researchers found that carotenoids are involved in blocking the cell cycle and inducing cell accumulation in the G0/G1 phase [66]. Recently, Gong et al. found that lutein significantly inhibited the growth of triple-negative breast cancer cells through cell cycle arrest [56]. G1/S, as a key checkpoint regulating DNA replication in the cell cycle, blocking the transition from the G1 phase to the S phase can lead to apoptosis. In addition to this, several studies have demonstrated that carotenoids can arrest the cell cycle in the G0/G1 phase through the suppression of cyclin D1 expression, consequently impacting the proliferation of cancer cells [67,68,69]. In conclusion, it is suggested that ROS production induced by carotenoid extracts (XY and ZH) leads to cell cycle arrest in the G0/G1 phase and apoptosis.

**Figure 9:**
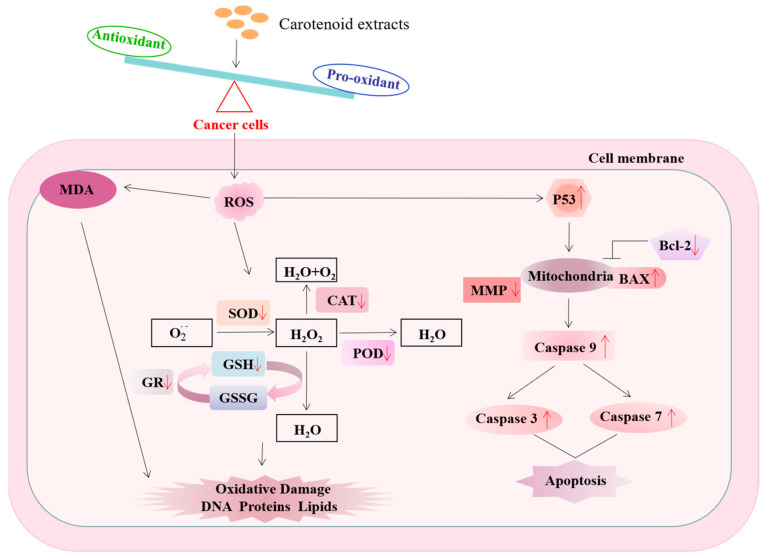
The schematic diagram represents the changes in the intracellular redox state and the expression of genes related to the endogenous apoptotic pathway induced by carotenoid extracts.

Intracellular ROS are key mediators of cancer cell proliferation and apoptosis through dysregulation of cellular homeostasis and are a marker of overall oxidative stress in cells [70]. After carotenoid extract treatment, we not only observed a concentration-dependent increase in ROS levels but also a significant decrease in MMP. According to previous studies, ROS generation is accompanied by an increase in p53 levels and the occurrence of apoptosis, forming a positive feedback loop that leads to a vicious cycle and further aggravates oxidative stress [71]. Elevated ROS may lead to excessive accumulation of ROS, triggering the mitochondrial phospholipid bilayer and subsequent reduction in MMP [72]. P53, a redox-active transcription factor, plays a crucial role in cellular responses to genotoxic stresses, including cell cycle arrest and apoptosis. Our study revealed a significant up-regulation in the gene expression of the tumor suppressor P53 with increasing concentrations of carotenoid extracts, as compared to the control. Xu et al. reported that P53 induces apoptosis by activating pro-apoptotic factors (BAX) and inhibiting anti-apoptotic factors (Bcl-2) [73]. Most chemotherapeutic drugs regulate apoptosis by modulating the permeability of the mitochondrial outer membrane, in which Bcl-2 and BAX family proteins are important proteins that play an anti-apoptotic and pro-apoptotic role [74,75]. In this study, carotenoid extracts up-regulated the mRNA level of *Bax* and down-regulated the expression of *Bcl-2*, and the ratio of *BAX*/*Bcl-2* was significantly increased, indicating that apoptosis occurred. Bcl-2 can selectively bind to Bax and block its polymerization reaction, destroy the permeability of the mitochondrial membrane, then activate the apoptosis promoter caspase-9, and further target the activation of apoptosis effector caspase-3 and caspase-7 [76,77]. Our results showed that carotenoid extracts up-regulated gene expression in the caspase cascade, including *caspase-9*, *caspase-3*, and *caspase-7*. These results indicate that carotenoid extracts induced cell apoptosis through the intrinsic apoptosis pathway. According to the above results, carotenoid extracts induced the accumulation of ROS in MDA-MB-231 cells, resulting in intracellular redox imbalance, blocked cell proliferation, and mitochondrial-dependent apoptosis (Figure 9).

## 5. Conclusions

In summary, the carotenoid-rich extracts extracted from citrus fruits have the effects of disrupting cell redox homeostasis, inhibiting cell proliferation, and inducing apoptosis in triple-negative breast cancer. Carotenoid extracts derived from XY and ZH induce ROS accumulation in cells due to their pro-oxidant effect, which aggravates oxidative stress and regulates apoptosis in MDA-MB-231 cells. Carotenoid extracts can activate the caspase cascade to induce cell apoptosis in a mitochondrial-dependent pathway, which may contribute to the sensitivity of cancer cells to drugs. Our research suggests the potential application value of citrus carotenoid extracts in breast cancer therapy. It is recommended that carotenoid-rich extracts be used as nutraceutical or chemotherapeutic drug sensitizers in cancer treatment.

## Figures and Tables

**Table 1:** Information on citrus materials.

No.	Citrus Resources	Latin Name	Abbreviation	Origin
1	ZaoMiPengGan	*Citrus reticulata* Blanco cv. Ponkan	ZMPG	Xiangxi (Hunan)
2	DongFangHong	*Citrus reticulata* Blanco cv. DongFangHong	DFHJ	Nanfeng (Jiangxi)
3	NanFengMiJu	*Citrus reticulata* Blanco cv. Kinokuni	NFMJ	Nanfeng (Jiangxi)
4	XiYou	*Citrus paradisi* Macf.	XY	South Africa
5	ZaoHongQiCheng	*Citrus sinensis* Osbeck cv. ‘ZaoHong’	ZH	Zigui (Hubei)

**Table 2:** Basic information of 9 carotenoids detected and identified in citrus pulp.

Name	Retention Time (min)	Peaks	Chemical Structure	Molecular Formula
lutein	20.34	445.7, 472.4	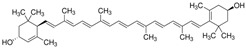	C_40_H_56_O_2_
Zeaxanthin	21.60	451.8, 475.9	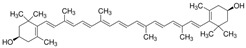	C_40_H_56_O_2_
β-cryptoxanthin	27.73	451.8, 479.7	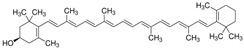	C_40_H_56_O
α-carotene	32.87	446.9, 474.8	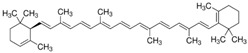	C_40_H_56_
β-carotene	35.11	453.0, 479.7	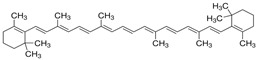	C_40_H_56_
Lycopene	55.16	473.7, 505.2	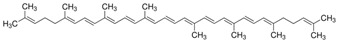	C_40_H_56_
violaxanthin	16.15	438.6, 467.5	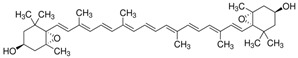	C_40_H_56_O_4_
9-cis-violaxanthin	18.36	436, 463.9	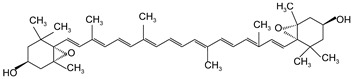	C_40_H_56_O_4_
Luteoxanthin	17.31	422.7, 448.1	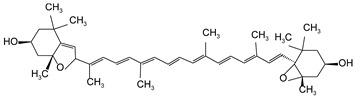	C_40_H_56_O_4_

Note: The information in the table is quoted from our published study [9].

**Table 3:** IC50 values (µg/mL) of citrus pulp carotenoid extracts in MDA-MB-231 cells.

Carotenoid Extracts’ Semi-Inhibitory Concentrations					
	DFHJ	NFMJ	ZH	ZMPG	XY
IC50 (µg/mL)	152.46	143.09	94.89	138.16	106.76

## Data Availability

All of the data is contained within the article and the Supplementary Materials.

## Supplementary Materials

The following supporting information can be downloaded at: https://www.mdpi.com/article/10.3390/antiox13030264/s1, Table S1: Information of gene primer sequences.
